# β-Lactam Resistance in Upper Respiratory Tract Pathogens Isolated from a Tertiary Hospital in Malaysia

**DOI:** 10.3390/pathogens10121602

**Published:** 2021-12-09

**Authors:** Soo Tein Ngoi, Anis Najwa Muhamad, Cindy Shuan Ju Teh, Chun Wie Chong, Kartini Abdul Jabar, Lay Ching Chai, Kin Chong Leong, Loong Hua Tee, Sazaly AbuBakar

**Affiliations:** 1 Department of Medical Microbiology, Faculty of Medicine, Universiti Malaya, Kuala Lumpur 50603, Malaysia; ngoisootein@um.edu.my (S.T.N.); anisnajwa@um.edu.my (A.N.M.); kartini.abduljabar@ummc.edu.my (K.A.J.); sazaly@um.edu.my (S.A.); 2 School of Pharmacy, Monash University Malaysia, Bandar Sunway 47500, Malaysia; chong.chunwie@monash.edu; 3 Faculty of Science, Institute of Biological Sciences, Universiti Malaya, Kuala Lumpur 50603, Malaysia; lcchai@um.edu.my; 4 Shionogi Singapore Pte Ltd., Anson Road, #34-14 International Plaza, Singapore 079903, Singapore; kinchong.leong@shionogi.com.sg (K.C.L.); loonghua.tee@shionogi.com.sg (L.H.T.); 5 Tropical Infectious Diseases Research and Education Centre (TIDREC), Universiti Malaya, Kuala Lumpur 50603, Malaysia

**Keywords:** antimicrobial resistance, β-lactamase, *Haemophilus influenzae*, penicillin resistance, penicillin-binding proteins, *Staphylococcus aureus*, *Streptococcus pneumoniae*

## Abstract

The rise of antimicrobial resistance (AMR) among clinically important bacteria, including respiratory pathogens, is a growing concern for public health worldwide. Common causative bacteria for upper respiratory tract infections (URTIs) include *Streptococcus* *pneumoniae* and *Haemophilus influenzae*, and sometimes *Staphylococcus aureus*. We assessed the β-lactam resistant trends and mechanisms of 150 URTI strains isolated in a tertiary care hospital in Kuala Lumpur Malaysia. High rates of non-susceptibility to penicillin G (38%), amoxicillin-clavulanate (48%), imipenem (60%), and meropenem (56%) were observed in *S. pneumoniae*. Frequent mutations at STMK and SRNVP motifs in PBP1a (41%), SSNT motif in PBP2b (32%), and STMK and LKSG motifs in PBP2x (41%) were observed in *S. pneumoniae*. *H. influenzae* remained highly susceptible to most β-lactams, except for ampicillin. Approximately half of the ampicillin non-susceptible *H. influenzae* harboured PBP3 mutations (56%) and only *bla*TEM was detected in the ampicillin-resistant strains (47%). Methicillin-susceptible *S. aureus* (MSSA) strains were mostly resistant to penicillin G (92%), with at least two-fold higher median minimum inhibitory concentrations (MIC) for all penicillin antibiotics (except ticarcillin) compared to *S. pneumoniae* and *H. influenzae*. Almost all URTI strains (88–100%) were susceptible to cefcapene and flomoxef. Overall, β-lactam antibiotics except penicillins remained largely effective against URTI pathogens in this region.

## 1. Introduction

Respiratory tract infections (RTIs) are generally classified into either upper or lower respiratory tract infections [1]. Based on national surveillance conducted by the Ministry of Health (MOH) Malaysia, RTIs accounted for approximately 68% of the patients admitted to government hospitals due to diseases of the respiratory system [2]. Among the RTIs, pneumonia is of particularly high risk to susceptible hosts in healthcare settings [3] and is the world’s leading infectious cause of death for infants under five, accounting for 16% of child deaths [4]. In Malaysia, pneumonia is one of the most common nosocomial infections, second only to clinical sepsis cases [5]. Common bacterial pathogens causing pneumonia are *Streptococcus pneumoniae, Haemophilus influenzae*, and sometimes *Staphylococcus aureus* (4). Non-viral pneumonia is and has been the main cause of death (99%) among patients’ death due to RTIs in Malaysian hospitals [6].

Antibiotic treatment is often contraindicated for healthy individuals with uncomplicated acute RTIs. However, in RTIs caused by bacteria, amoxicillin or penicillin antibiotics are often the drugs of choice, followed by macrolides or fluoroquinolones in cases of penicillin allergy [4,7,8]. In Malaysia, higher rates of antibiotic prescription for upper respiratory tract infections (URTIs) were observed in private clinics (46.7%) compared to public clinics (27.8%) [9]. Most of the antibiotics prescribed were penicillin and macrolides, with broad-spectrum antibiotics being the preferred option [9]. The higher-than-usual antibiotic prescription rates for URTIs, which are mainly caused by viruses, may increase the risk of bacterial pathogens developing antimicrobial resistance (AMR) [9]. The first-line drugs that are commonly used for the treatment of acute RTIs mainly comprise β-lactam antibiotics [4,7,8]. However, β-lactam resistance among the clinically important bacteria, including those that cause RTIs, has been on a global rise [10]. Therefore, continuous efforts to monitor the AMR trends and to explore new antibacterial agents are essential to reduce the impact of AMR on public health [11]. This study aimed to investigate the β-lactam resistance trends and mechanisms among *S. pneumoniae, H. influenzae,* and *S. aureus* strains isolated from URTI patients.

## 2. Results

The patient’s demographic data and *S. pneumoniae* serotypes are summarized in Table 1. Table 2 summarizes the minimum inhibitory concentration (MIC) data for all 150 bacterial strains.

### 2.1. Streptococcus Pneumoniae

**Figure 1:**
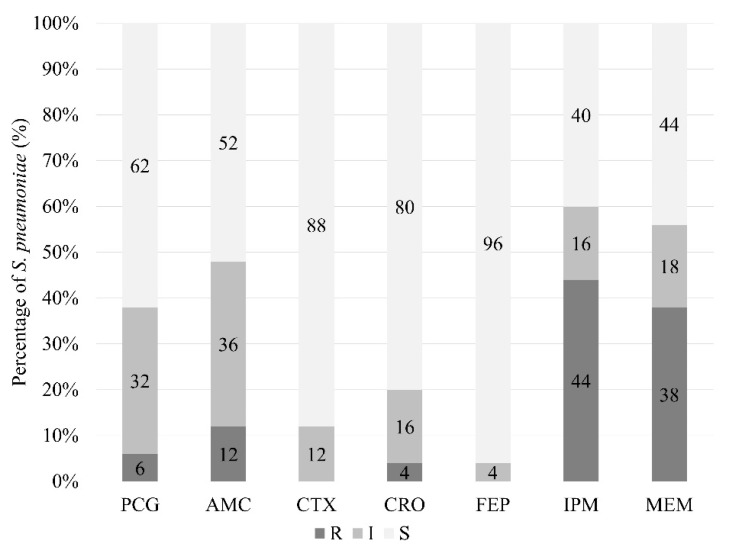
Proportions of *S. pneumoniae* strains (*n* = 50) with different susceptibility phenotypes. The MIC values for penicillin G (PCG), amoxicillin-clavulanate (AMC), cefotaxime (CTX), ceftriaxone (CRO), cefepime (FEP), imipenem (IPM), and meropenem (MEM) were interpreted based on the Clinical and Laboratory Standards Institute (CLSI) non-meningitis breakpoints.

*S. pneumoniae* showed the highest rates of resistance to imipenem (44%) and meropenem (38%) (Figure 1). Among the two major *S. pneumoniae* serotypes observed in this study, serotype 19F showed higher rates of non-susceptibility to penicillin (55%), imipenem (91%), and meropenem (91%) compared to serotype 6A/6B (36%, 55%, 45%, respectively). The potential non -susceptibility to ampicillin was predicted in 74% of the *S. pneumoniae* strains (penicillin MIC > 0.06 µg/mL) [8]. Simultaneous resistance to two-or-more β-lactam antibiotics was observed in 34% of the strains (Supplementary Table S1).

High-level resistance (MIC_50_ ≥ 64 µg/mL) to ticarcillin, ticarcillin-clavulanate, and cefoxitin was observed among the *S. pneumoniae* strains (Table 2). Penicillin antibiotics showed relatively higher MIC_50_ values (2–≥ 64 µg/mL), compared to other groups of β-lactam. Generally, β-lactam combination agents remained highly active against *S. pneumoniae* (MIC_50_ ≤ 0.5 µg/mL), except amoxicillin-clavulanate and ticarcillin-clavulanate. Similarly, most third and fourth-generation cephalosporins (cefcapene, cefotaxime, ceftriaxone, and cefepime) and the oxacephem antibiotic flomoxef recorded low MIC_50_ values (≤0.5 µg/mL). Cefotaxime-clavulanate showed highest activity against the *S. pneumoniae* (MIC_90_ = 0.5 µg/mL).

Thirty-seven ampicillin non-susceptible *S. pneumoniae* strains were examined for amino acid substitutions in three conserved motifs of the *pbp* genes (Table 3). Seven strains failed to produce amplicons for all three *pbp* genes, probably due to primer-binding sites alteration. The KTG motif in the penicillin-binding protein PBP1a and SVVK in PBP2b remained highly conserved. All but five mutation profiles (M01, M07, M09, M11, and M14) conferred reduced susceptibility to penicillin G (MIC ≥ 4 µg/mL). M12 and M13 profiles were penicillin-resistant *S. pneumoniae* (PRSP). Mutations beyond the conserved motifs were identified in PBP1a (block substitution of Thr-Ser-Gln-Phe (T^574^SQF) by Asn-Thr-Gly-Tyr (N^574^TGY); *n* = 16) and PBP2b (Ser-411-Pro, Asn-421-Tyr, Thr-425-Lys, and Gln-426-Ala/Leu; *n* = 11).

### 2.2. Haemophilus Influenzae

The majority of the *H. influenzae* strains were resistant to ampicillin (72%) and non-susceptible to ceftazidime (20%), amoxicillin-clavulanate (18%), cefotaxime (6%), and imipenem (6%). All strains remained susceptible to piperacillin-tazobactam, ceftriaxone, cefepime, and meropenem. Simultaneous resistance/non-susceptibility to two to three β-lactam antibiotics was observed in approximately one-third of the strains (*n* = 16) (Supplementary Table S2).

High-level resistance was not common among the *H. influenzae* strains (Table 2). The MIC_50_ values of amoxicillin-clavulanate, piperacillin-tazobactam, cefotaxime, ceftazidime, ceftriaxone, cefepime, imipenem, and meropenem were within the susceptible range (Clinical and Laboratory Standards Institute, CLSI interpretive criteria). Cefmetazole (MIC_50_ = 8 µg/mL), ampicillin (MIC_50_ = 4 µg/mL), and cefoxitin (MIC_50_ = 4 µg/mL) were relatively less active against the *H. influenzae* strains. The majority of the β-lactam combination agents, third and fourth generation cephalosporins, piperacillin, and meropenem remained highly active against *H. influenzae* (MIC_50_ ≤ 0.25 µg/mL). *H. influenzae* were mostly inhibited by low concentrations of cefotaxime-clavulanate, cefcapene, cefotaxime, ceftriaxone, cefepime, and meropenem (MIC_90_ ≤ 0.5 µg/mL). Contrarily, high concentrations of penicillin were required to inhibit the growth of most of the *H. influenzae* strains (MIC_90_ > 64 µg/mL).

Thirty-nine ampicillin non-susceptible *H. influenzae* strains were examined for β-lactamase (*bla*) genes and *ftsI* mutations (Table 4). The *bla*_TEM_ gene was detected in 44% (*n* = 17) of the strains, while *bla*_ROB_ was totally absent. The *bla*_TEM_-positive strains were further classified as β-lactamase positive ampicillin-resistant (BLPAR; harbours wild type *ftsI* gene) (*n* = 7) or β-lactamase positive amoxicillin-clavulanate resistant (BLPACR; harbours *ftsI* gene mutation) (*n* = 10). Approximately one-third (31%) of the ampicillin-resistant strains lacked β-lactamase but harboured a mutated *ftsI* gene (β-lactamase negative ampicillin-resistant, BLNAR). The amino acid substitutions in the *ftsI* gene were generally more diverse in BLNAR strains than in BLPACR strains.

### 2.3. Methicillin-Susceptible Staphylococcus aureus

Susceptibility to oxacillin (MIC ≤ 2 µg/mL) and cefoxitin (MIC ≤ 4 µg/mL) confirmed the methicillin-susceptible phenotype of the *S. aureus* strains (Table 2). The majority of the methicillin-susceptible *S. aureus* (MSSA) strains showed a penicillin-resistant phenotype (92%). Higher MIC values of penicillinase-labile agents such as ampicillin, piperacillin, and ticarcillin (16 ≤ MIC_50_ ≤ 64 µg/mL) were observed among penicillin-resistant MSSA. High MIC values were recorded for ceftazidime (MIC_50_ = 128 µg/mL) and ceftazidime-clavulanate (MIC_50_ = 64 µg/mL). Carbapenems (MIC_50_ ≤ 0.06 µg/mL) generally had lower MIC_50_ values than β-lactam combination agents (1 ≤ MIC_50_ ≤ 64 µg/mL) and cephems (1 ≤ MIC_50_ ≤ 128 µg/mL), except cefcapene. Most MSSA strains remained highly susceptible to carbapenems, cefcapene and flomoxef (MIC_90_ ≤ 1 µg/mL).

### 2.4. In Vitro Efficiency of Flomoxef and Cefcapene

**Figure 2:**
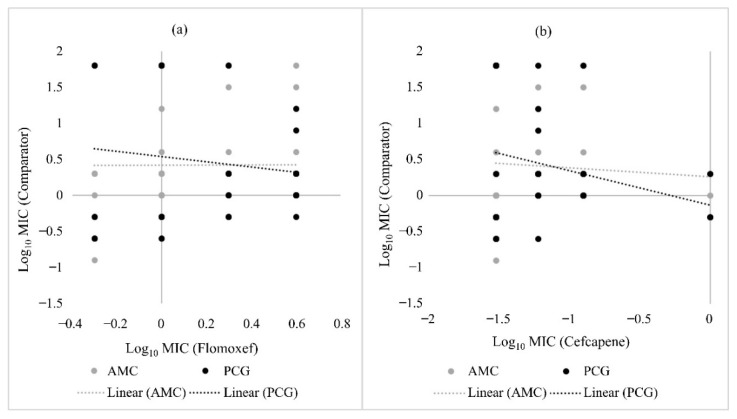
Scatter plots comparing the in vitro activity of amoxicillin-clavulanate, penicillin G, flomoxef and cefcapene against *H. influenzae*. (**a**) compares the Log_10_ MIC values of amoxicillin-clavulanate (AMC) and penicillin G (PCG) against that of flomoxef, while (**b**) compares the Log_10_ MIC values of AMC and PCG against that of cefcapene. The dotted lines indicate the linear correlation between the comparators.

**Figure 3:**
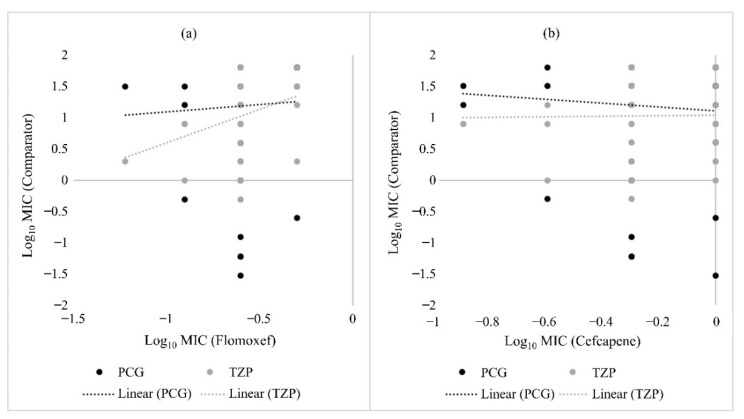
Scatter plots comparing the in vitro activity of penicillin G, piperacillin-tazobactam, flomoxef and cefcapene against MSSA. (**a**) compares the Log_10_ MIC values of penicillin G (PCG) and piperacillin-tazobactam (TZP) against flomoxef, while (**b**) compares the Log_10_ MIC values of PCG and TZP against cefcapene. The dotted lines indicate the linear correlation between the comparators.

**Figure 4:**
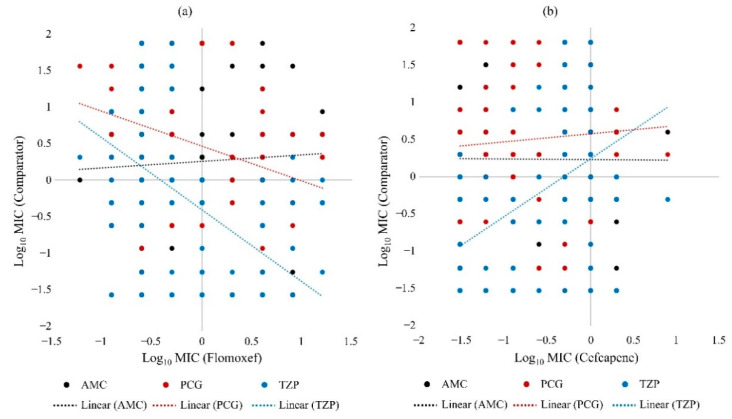
Scatter plots comparing minimum inhibitory concentration (MIC) values of selected β-lactam antibiotics for 150 bacterial strains. (**a**) compares the MIC values of amoxicillin-clavulanate (AMC), penicillin G (PCG) and piperacillin-tazobactam (TZP) against that of flomoxef, while (**b**) compares the MIC values of AMC, PCG and TZP against that of cefcapene. The dotted lines indicate the linear correlation between the comparators.

The susceptibility of *H. influenzae* to penicillin G was negatively correlated with both flomoxef and cefcapene, while that of amoxicillin-clavulanate was only negatively correlated with cefcapene (Figure 2). In MSSA, susceptibility to penicillin G was positively correlated with flomoxef (Figure 3a) but negatively correlated with cefcapene (Figure 3b). Similarly, a positive relationship was only observed between piperacillin-tazobactam and flomoxef in MSSA (Figure 3). For *S. pneumoniae* strains, none of the β-lactam antibiotics selected for comparison showed an obvious correlation with both flomoxef and cefcapene (data not shown). An overall comparison including all URTI strains revealed negative correlations between penicillin G and piperacillin-tazobactam with flomoxef, but the opposite with cefcapene (Figure 4).

A cross-species comparison between flomoxef and cefcapene showed that both antimicrobial agents inhibited the growth of more than half of the organisms at very low concentrations (Table 5). Flomoxef was most active against MSSA (MIC ≤ 1 µg/mL) while cefcapene was most active against *H. influenzae* (MIC ≤ 1 µg/mL). By using referral MIC breakpoints, all *H. influenzae* and MSSA strains were predicted as susceptible to both flomoxef and cefcapene. Most of the *S. pneumoniae* strains showed susceptible phenotypes for cefcapene (98%) and flomoxef (88%).

## 3. Discussion

A total of 150 bacterial strains comprised of three common URTI organisms were examined. All *H. influenzae* and *S. pneumoniae* were isolated from paediatric patients, while MSSA was isolated from patients from a wider age range. This sampling outcome was not unexpected as both *H. influenzae* and *S. pneumoniae* are the two most common causative agents for RTIs in children. In Malaysia, the Hib conjugate vaccine for *H. influenzae* has been included in the National Immunization Programme. Pneumococcal vaccine was not listed among the mandatory vaccines in our country. Despite the implementation of these vaccination programmes, the rates of *S. pneumoniae* and *H. influenzae* remain high among children and cause more severe infections. Due to the high prevalence and reduced susceptibility of *S. pneumoniae* and *H. influenzae* strains isolated from children worldwide, therefore we focused on the AMR trends and mechanisms of these two organisms in paediatric patients for this study [15,16,17]. Contrarily, paediatric RTIs caused by MSSA are less frequent. From the year 2012 to 2015, a total of 7434 *S. aureus* infections had been reported in UMMC and 76.6% of the cases were caused by MSSA, among which only 22.2% involved paediatric patients (unpublished data). This record shows that although MSSA infections occurred more frequently than its methicillin-resistant counterpart, it is somehow less studied. Therefore, there was limited AMR data for MSSA in this region.

The relatively high rates of non-susceptibility to penicillin, amoxicillin-clavulanate and carbapenems in *S. pneumoniae* are a cause of public health concern among children. Previous studies conducted in the Southeast Asian region have documented relatively high rates of penicillin non-susceptibility (>50%) among paediatric populations [18,19,20]. Our findings indicated that *S. pneumoniae* serotype 19F was commonly associated with non-susceptibility to penicillin and carbapenem resistance, in agreement with the previous notion that this serotype often shows greater AMR tendency [18,19,21].

We observed the most frequent mutations at Thr-371-Ala/Ser (STMK motif) and Pro-432-Thr (adjacent to SRN motif) in PBP1a, corresponding to the common mutation sites in penicillin non-susceptible *S. pneumoniae* [22,23]. The block mutation identified at amino acid positions 574–577 in PBP1a has been previously associated with intermediate- to high-level penicillin resistance [24,25]. The most frequent mutation in PBP2b occurred at Thr-445-Ala (adjacent to the SSN motif). All strains with Thr-445-Ala substitution showed penicillin MIC ≥ 0.25 µg/mL, consistent with a recent work reporting this mutation to reduce PBP binding affinity by 60% and resulting in raised penicillin MIC (>0.25 µg/mL) [26]. Additional mutations beyond the active sites of PBP2b observed in this study had been reported to decrease β-lactams reactivity and is commonly present in strains with penicillin MIC > 0.5 µg/mL [27]. The most common mutations in PBP2x (Thr-338-Ala in STMK motif and Leu-546-Val adjacent to KSG motif) were identified in *S. pneumoniae* strains exhibiting raised penicillin MICs (2–8 µg/mL), consistent with previous reports associating these mutations with higher-level β-lactam resistance [28,29,30]. Similar to other non-susceptible *S. pneumoniae* reported globally, His-395-Leu adjacent to the SSN motif in PBP2x was not associated with Thr-338-Ala (in STMK motif) and conferred a lower penicillin MIC (1 µg/mL) [28]. The only strain harbouring Met-339-Phe alongside Thr-338-Ala in the STMK motif of PBP2x did not show high penicillin and cefotaxime MIC values (2 and 0.5 µg/mL, respectively), although mutation at this site was associated with drastically decreased acylation efficiency of β-lactams and results in higher penicillin and cefotaxime MICs (≥4 and ≥2 µg/mL, respectively) [30,31,32]. The combined effect of the mutations at all three PBPs has resulted in the raised penicillin MICs (≥0.25 µg/mL) observed in this study, thus accounting for the high MIC_50/90_ values of ticarcillin, ticarcillin-clavulanate and cefoxitin which are known to be less active against penicillin-resistant *S. pneumoniae* [33,34].

*H. influenzae* remained highly susceptible to most of the β-lactam antibiotics tested, except ampicillin and cephamycin. The high rate of ampicillin resistance among the URTI strains observed in this study could be the combined effect of β-lactamase production and penicillin-binding protein 3 (PBP3) alteration. The high prevalence of BLNAR and BLPACR in this study indicates that PBP3 alteration resembles the main resistance mechanism against ampicillin in local *H. influenzae* strains. This is noteworthy since β-lactamase production has been the main ampicillin resistance mechanism for *H. influenzae* globally except Japan [35]. The majority of the PBP3-altered strains in our study were classified as subgroups II, with mutations that conferred only low-level β-lactam resistance (low-rPBP3) [13,36]. This observation concurred with the previous notion that low-rPBP3 predominates in *H. influenzae* isolated in most parts of the world except Japan and South Korea [37,38]. The high prevalence of PBP3 alteration among our ampicillin-resistant *H. influenzae* strains could have explained the reduced susceptibility to cephamycin since PBP3 mutations are also known to confer non-susceptibility to second and third-generation cephalosporins [38].

The high rate of penicillin resistance among the MSSA strains in this study reflects the common AMR trends of *S. aureus* in Malaysia. Indeed, the national surveillance data recorded a consistently high rate of penicillin resistance (≥80%) among the clinical *S. aureus* isolated yearly [39]. This is not surprising given the high prevalence of the *bla*Z gene in local *S. aureus* populations, including those associated with nasal carriage [40]. The higher MIC values for penicillinase-labile penicillin (ampicillin, piperacillin, and ticarcillin) among the MSSA strains are common in penicillin-resistant but oxacillin-susceptible *S. aureus* [41]. Ceftazidime (with or without clavulanate) showed high MIC values, which is a predictable observation since ceftazidime is known to be less active against *S. aureus* [42]. The higher activity of penicillinase-stable agents, including the β-lactam combination agents (except ceftazidime-clavulanate and piperacillin-tazobactam), cephems (except ceftazidime), and carbapenems are expected in MSSA [43]. In contrast with *mec*A-mediated resistance, *bla*Z-mediated penicillin resistance in *S. aureus* rarely produces an effect on other β-lactam antibiotics even at high inoculum size [44,45,46].

Flomoxef and cefcapene, currently not available in Malaysia, showed well in vitro activity against the URTI pathogens examined in this study. Both antibiotics were highly effective against *H. influenzae* that were non-susceptible to penicillin and amoxicillin-clavulanate. Furthermore, cefcapene showed greater activity against penicillin-resistant MSSA, while flomoxef was highly effective against URTI strains with reduced susceptibility to penicillin and piperacillin-tazobactam. Both cefcapene (third-generation cephalosporin) and flomoxef (previously a fourth-generation cephalosporin but currently grouped in oxacephem (cephalosporin group IIIC)) exhibit broad-spectrum activity against Gram-positive and Gram-negative bacteria [47]. However, third-generation cephalosporins are more active against Gram-negative bacteria especially *Enterobacteriaceae*, *Neisseria* spp. and *H. influenzae* [48]. Indeed, cefcapene showed lower MIC_50/90_ values in *H. influenzae* compared to *S. pneumoniae* and MSSA in this study. Although fourth-generation cephalosporins are similar to the third generation, members of this group possess zwitterionic compounds that penetrate through the outer membrane of Gram-negative bacteria more rapidly [49]. Furthermore, fourth-generation cephalosporins show a higher affinity to PBPs compared to β-lactamase in both Gram-positive and Gram-negative organisms [50]. Our results showed that the PBP alterations that conferred penicillin resistance might not be effective against flomoxef, hence the negative correlation between the susceptibility of these two agents among the URTI organisms.

In short, both cefcapene and flomoxef were highly active against bacterial strains that were increasingly resistant to β-lactams commonly used to treat URTIs and pneumonia. Based on the Malaysian National Antimicrobial Guideline 2019, penicillin is recommended for the treatment of URTIs, while amoxicillin-clavulanate is recommended for the treatment of both upper and lower RTIs [51]. However, our study shows increased non-susceptibility to penicillin and amoxicillin-clavulanate among the common URTI pathogens. The MIC_50/90_ values of flomoxef reported in this study were comparable with, sometimes slightly lower than, similar studies conducted in China and Japan, indicating its higher activity against local URTI strains [52,53,54]. Similar to findings reported in Korea, cefcapene showed lower MIC_50/90_ values compared to other cephalosporins [55]. Both cefcapene and flomoxef have been clinically proven to be effective and well-tolerated in patients with RTIs [56,57,58,59].

Although our study was a single-site study that involved only small subsets of strains, the data provided herein may improve current knowledge on the extent of non-susceptibility to β-lactams and the associated resistance mechanisms in local URTI pathogens. Nonetheless, multiple centres and larger sample sizes should be included in future studies to provide a more comprehensive understanding of the β-lactam resistance trends and mechanisms among the important URTI pathogens in this region. Another limitation of the current study was that only selected genes encoding for penicillin-binding proteins (*pbp* and *fts*I) and β-lactamases (*bla*_TEM_ and *bla*_ROB_) were investigated. Although these genes represent the most common β-lactam resistance mechanisms in *S. pneumoniae* and *H. influenzae*, other molecular mechanisms could have been accounted for the raised MIC values observed in the strains that lacked these genes (or mutations).

## 4. Materials and Methods

### 4.1. Bacterial Strains

A total of 150 non-duplicated bacterial strains comprised of *S. pneumoniae* (*n* = 50), *H. influenzae* (*n* = 50), and methicillin-susceptible *S. aureus* (MSSA) (*n* = 50) were revived from the bacterial stock cultures collection in the diagnostic laboratory of University Malaya Medical Centre (UMMC). All strains were isolated from respiratory tract specimens of patients admitted to the UMMC with URTI from 2013 to 2015. Both *H. influenzae* and *S. pneumoniae* were collected from only paediatric patients (aged 0–17) while *S. aureus* was collected from patients of all age groups. All bacterial strains were isolated from bronchoalveolar lavage, nasopharyngeal swab and sputum specimens of the patients. Bacterial strains isolated from other sites were excluded from the study. The isolation and initial identification of the bacterial strains were part of the routine microbiological examination procedures in the hospital’s diagnostic laboratory. The identity of the bacterial strains was further confirmed using polymerase chain reaction (PCR) protocols adapted from published studies [43,60,61]. *S. pneumoniae* strains were further subjected to PCR serotyping using previously described protocols [62].

### 4.2. Minimum Inhibitory Concentrations and Comparison of In Vitro Activity

The MICs of twenty β-lactam antibiotics were determined using the broth microdilution method based on CLSI guidelines [41]. The antimicrobial agents examined in this study include penicillins (ampicillin, penicillin G, piperacillin, and ticarcillin), β-lactam combination agents (amoxicillin-clavulanate, cefotaxime-clavulanate, ceftazidime-clavulanate, piperacillin-tazobactam, and ticarcillin-clavulanate), cephems (cefmetazole, cefoxitin, cefcapene, cefoperazone, cefotaxime, ceftazidime, ceftriaxone, cefepime, and flomoxef) and carbapenems (imipenem and meropenem). The methicillin susceptibility of the *S. aureus* was further confirmed via determining the MIC of oxacillin in addition to the β-lactams aforementioned. The MIC values of the antimicrobial agents were interpreted according to the available breakpoints in CLSI guidelines [41]. The median MIC value for each antimicrobial agent is represented as MIC_50_, and MIC_90_ indicates a concentration that effectively inhibited 90% of the tested strains. Scatter plots were generated using log_10_ MIC values to compare the activity of flomoxef and cefcapene against the common β-lactam antibiotics recommended for the treatment of RTIs based on the Malaysian National Antibiotics Guideline [51].

### 4.3. Molecular Detection of Penicillin Resistance-Conferring Genes

*S. pneumoniae* and *H. influenzae* strains with non-susceptible phenotypes for ampicillin were examined for potential amino acid substitution in the genes encoding for penicillin-binding proteins (pbp1a, pbp2b, and pbp2x in *S. pneumoniae* and *ftsI,* also known as pbp3, in *H. influenzae*). The primer sequences and PCR conditions for the amplification of *pbp* and *ftsI* genes were adapted from published studies [12,22,63]. The PCR products were then purified using MEGAquick-spin™ Plus Total Fragment DNA Purification Kit (iNtRON Biotechnology, South Korea), and sent for sequencing by a commercial sequencing service provider (First BASE Laboratories, Malaysia). The DNA sequences obtained were then analysed using MEGA-X software [64].

The presence of β-lactamase genes *bla*_TEM_ (526 bp) and *bla*_ROB_ (692 bp) in the *H. influenzae* strains were investigated using PCR primers and reaction-mix content adapted from a published study [65].

## 5. Conclusions

In conclusion, URTI-associated *S. pneumoniae*, *H. influenzae* and MSSA remained largely susceptible to most of the β-lactams but showed increased non-susceptibility to penicillin antibiotics. High-level ampicillin resistance in *H. influenzae* was mainly mediated by the *bla*_TEM_ gene. Multiple-sites mutation in the PBPs was responsible for the penicillin non-susceptible phenotypes of *S. pneumoniae* and *H. influenzae*. Cefcapene and flomoxef showed comparable in vitro activity with cephalosporins and carbapenems, thus could be considered as alternative options for the empirical treatment of URTIs.

## Figures and Tables

**Table 1:** Summary of demographic data, source of specimen and serotype of the URTI strains (*n* = 150).

	*S. pneumoniae* (*n*) (%)	*H. influenzae* (*n*) (%)	MSSA (*n*) (%)
**Gender**			
Female	27 (54)	21 (42)	18 (36)
Male	23 (46)	29 (58)	32 (64)
**Age Range**			
<1 year	7 (14)	5 (10)	12 (24)
1–17	43 (86)	45 (90)	10 (20)
18–59	0 (0)	0 (0)	18 (36)
≥60	0 (0)	0 (0)	10 (20)
**Source of Specimen**			
Bronchoalveolar lavage	2 (4)	0 (0)	0 (0)
Nasopharyngeal swab	46 (92)	50 (100)	50 (100)
Sputum	2 (4)	0 (0)	0 (0)
**Serotype ^1^**			
3	1 (2)	-	-
34	1 (2)	-	-
11A/D/F	3 (6)	-	-
15A/F	1 (2)	-	-
19A	2 (4)	-	-
19F	11 (22)	-	-
23A	3 (6)	-	-
23F	2 (4)	-	-
6A/6B	11 (22)	-	-
6C	3 (6)	-	-
Non-typeable	12 (24)	-	-

^1^ Polymerase chain reaction (PCR) serotyping was only performed on *S. pneumoniae* strains (*n* = 50).

**Table 2:** Summary of MIC data for *S. pneumoniae* (*n* = 50), *H. influenzae* (*n* = 50), and methicillin-susceptible *S. aureus* (MSSA) (*n* = 50).

Antimicrobial Agent	*S. pneumoniae* ^1^			*H. influenzae* ^1^			MSSA ^1^		
	MIC Range	MIC_50_	MIC_90_	MIC Range	MIC_50_	MIC_90_	MIC Range	MIC_50_	MIC_90_
Ampicillin	≤0.125–128	16	64	0.5–>256	4	>256	≤0.125–128	32	64
Penicillin G	≤0.03–8	2	4	0.25–>64	1	>64	≤0.03–>64	32	>64
Piperacillin	≤0.03–16	2	8	≤0.03–>64	≤0.03	>64	0.5–>128	64	>128
Ticarcillin	1–>64	≥64	>64	0.25–>64	2	>64	2–32	16	32
Amoxicillin-clavulanate (2:1)	≤0.03/0.015–32/16	2/1	8/4	0.125/0.06–>64/32	2/1	32/16	≤0.125/0.06–8/4	2/1	4/2
Cefotaxime-clavulanate ^2^	≤0.03–1	0.06	0.5	≤0.03–0.5	≤0.03	0.25	0.25–2	1	2
Ceftazidime-clavulanate ^2^	≤0.03–32	0.125	2	≤0.03–4	0.25	2	16–256	64	128
Piperacillin-tazobactam ^3^	≤0.03–2	0.5	1	≤0.03–1	≤0.03	1	0.5–64	16	64
Ticarcillin-clavulanate ^4^	1–>64	≥ 64	>64	0.125–16	1	2	0.5–16	8	8
Cefmetazole	0.125–64	4	32	1–8	8	8	0.5–2	1	2
Cefoxitin	32–>64	>64	>64	1–>64	4	>64	2–4	4	4
Cefcapene	≤0.03–8	0.5	2	≤0.03–1	≤0.03	0.125	0.125–1	0.5	1
Cefoperazone	≤0.03–32	4	32	≤0.03–16	0.125	8	0.25–8	4	8
Cefotaxime	≤0.03–2	0.125	2	≤0.03–4	≤0.03	0.25	0.25–2	1	2
Ceftazidime	≤0.03–64	2	16	0.06–64	1	8	32–>256	128	256
Ceftriaxone	≤0.03–4	0.5	2	≤0.03–0.5	≤0.03	0.25	2–4	2	4
Cefepime	≤0.03–2	0.25	1	0.06–2	0.25	0.5	0.5–4	2	2
Imipenem	0.06–32	0.5	16	0.5–32	2	4	≤0.03	≤0.03	≤0.03
Meropenem	≤0.03–2	0.5	1	≤0.03–0.5	0.125	0.5	≤0.03–0.06	0.06	0.06
Flomoxef	0.125–16	0.5	16	0.5–4	1	4	0.06–0.5	0.25	0.25
Oxacillin ^5^	-	-	-	-	-	-	≤0.125–0.5	0.25	0.5

^1^ All MIC values are indicated in µg/mL; ^2^ Fixed concentration of clavulanate (4 µg/mL); ^3^ Fixed concentration of tazobactam (4 µg/mL); ^4^ Fixed concentration of clavulanate (2 µg/mL); ^5^ Broth microdilution assay was only performed for MSSA strain.

**Table 3:** Mutations identified in the penicillin-binding proteins (PBPs) in *S. pneumoniae*.

Mutation Profile ^1^	No. of Strains	Ampicillin MIC Range (µg/mL)	Penicillin G MIC Range (µg/mL)	Penicillin-Binding Protein (PBP) motifs ^2,3^								
				PBP1a			PBP2b			PBP2x		
				STMK (370–373)	SRNVP (428–432)	KTG (557–559)	SVVK (385–388)	SSNT (442–445)	KTGTA (614–618)	STMK (337–340)	HSSN (395–397)	LKSG (546–549)
M01	1	4	1	----	-----	---	----	----	-----	----	L---	V---
M02	3	16–64	4	-S--	----T	---	----	----	-----	-A--	----	V---
M03	3	<0.125–64	1–4	-S--	----T	---	----	----	-----	----	----	----
M04	1	16	4	----	-----	---	----	---A	----G	-A--	----	V---
M05	3	16–32	4	-A--	----T	---	----	---A	-----	----	----	----
M06	3	4–32	2–4	-S--	----T	---	----	---A	-----	-A--	----	V---
M07	1	2	0.25	----	-----	---	----	---A	-----	----	----	----
M08	2	4–16	0.25–4	----	-----	---	----	---A	-----	-A--	----	V---
M09	2	2–32	0.5–2	----	-----	---	----	----	-----	-A--	----	V---
M10	1	16	4	-A--	----T	---	----	----	-----	----	----	----
M11	1	16	2	-A--	----T	---	----	---A	-----	-A--	----	V---
M12	1	64	8	-S--	----T	---	----	---A	----G	-A--	----	V---
M13	1	128	8	----	-----	---	----	----	-----	-A--	----	----
M14	1	32	2	----	-----	---	----	----	-----	-AF-	----	V---
$	6	<0.125–16	0.125–4	----	-----	---	----	----	-----	----	----	----

^1^ Arbitrarily designated mutation profiles; $: Absence of amino acid substitution at all sites. ^2^ The wild-type gene sequences from *S. pneumoniae* R6 (NCBI GenBank accession number: NC_003098) were used as the reference for mutation analysis. ^3^ A: Alanine; F: Phenylalanine; G: Glycine; H: Histidine; K: Lysine; L: Leucine; M: Methionine; N: Asparagine; P: Proline; R: Arginine; S: Serine; T: Threonine; V: Valine.

**Table 4:** Summary of *ftsI* mutation sites and classification of ampicillin-resistant *H. influenzae*.

Amino Acid Substitution Sites ^1,2^	Group ^3^	Ampicillin MIC Range (µg/mL)	No. (%) of Strains
**BLNAR**			
Asp-350-Asn; Met-377-Ile; Gly-490-Glu; Ala-502-Val; Asn-526-Lys	IIb	8	1 (9)
Asp-350-Asn; Gly-490-Glu; Ala-502-Val; Asn-526-Lys	IIb	2–4	5 (46)
Asp-350-Asn; Gly-490-Glu; Asn-526-Lys; Ala-530-Ser	IIa	4	2 (18)
Asp-350-Asn; Met-377-Ile; Ala-502-Val; Asn-526-Lys	IIb	4	1 (9)
Asn-526-Lys; Ala-530-Ser	IIa	8–128	2 (18)
Asp-350-Asn	M	>256	1 (9)
**BLPACR**			
Asp-350-Asn; Gly-490-Glu; Asn-526-Lys; Ala-530-Ser	IIa	128	1 (9)
Ile-449-Val; Asn-526-Lys	IId	4–>256	3 (27)
Asp-350-Asn	M	4–>256	6 (55)

^1^ BLNAR: β-lactamase negative ampicillin-resistant strain; BLPACR: β-lactamase positive amoxicillin-clavulanate resistant; Ala: Alanine; Asp: Aspartic acid; Asn: Asparagine; Glu: Glutamic acid; Gly: Glycine; Ile: Isoleucine; Lys: Lysine; Met: Methionine; Ser: Serine; Val: Valine; M: Miscellaneous group. ^2^ The wild-type sequence of *fts*I gene from *H. influenzae* Rd KW20 (L42023) was used as the reference for mutation analysis. ^3^ Classification of strains based on key mutation sites [12,13,14].

**Table 5:** Comparison of flomoxef and cefcapene minimum inhibitory concentration values among different bacterial species.

Antimicrobial Agent	MIC Range (µg/mL)	Susceptibility Phenotype	*S. pneumoniae* (*n*) (%)	*H. influenzae* (*n*) (%)	MSSA (*n*) (%)
Flomoxef ^1^	≤1	Susceptible	29 (58)	25 (50)	50 (100)
	2–8	Susceptible	15 (30)	25 (50)	0 (0)
	≥16	Resistant	6 (12)	0 (0)	0 (0)
Cefcapene ^2^	≤1	Susceptible	40 (80)	50 (100)	50 (100)
	2	Intermediate	9 (18)	0 (0)	0 (0)
	≥4	Resistant	1 (2)	0 (0)	0 (0)

^1^ The MIC breakpoints for moxalactam (CLSI) were used as the reference for interpretive criteria of flomoxef. ^2^ The MIC breakpoints for ceftriaxone (CLSI) were used as the reference for interpretive criteria of cefcapene.

## Data Availability

The datasets used and/or analysed during the current study are available from the corresponding author on reasonable request.

## Supplementary Materials

The following are available online at https://www.mdpi.com/article/10.3390/pathogens10121602/s1, Table S1: Antimicrobial resistance (AMR) profiles of *S. pneumoniae* strains (*n* = 50); Table S2: Antimicrobial resistance (AMR) profiles of *H. influenzae* strains (*n* = 50).
