# Supplementary material for: β-Lactam Resistance in Upper Respiratory Tract Pathogens Isolated from a Tertiary Hospital in Malaysia
Source: Pathogens. 2021 Dec 9;10(12):1602. doi: 10.3390/pathogens10121602 (PMC8705930; doi:10.3390/pathogens10121602)
Supplement: Supplementary file 1 [file pathogens-10-01602-s001.zip › pathogens-1455523 supplementary.pdf]

Supplementary Table S1. Antimicrobial resistance (AMR) profiles of *S. pneumoniae* strains (n = 50).

AMR profile *	(*n*) (%)
PCG, AMC, CRO, IPM, MEM	1 (2)
PCG, AMC, IPM, MEM	1 (2)
AMC, IPM, MEM	2 (4)
IPM, MEM	13 (26)
AMC	2 (4)
CRO	1 (2)
IPM	5 (10)
MEM	2 (4)
PCG	1 (2)
$	22 (44)

* PCG: penicillin G; AMC: amoxicillin-clavulanate; CRO: ceftriaxone; IPM: imipenem; MEM: meropenem; $: susceptible to penicillin G, amoxicillin-clavulanate, cefotaxime, ceftriaxone, cefepime, imipenem and meropenem.

Supplementary Table S2. Antimicrobial resistance (AMR) profiles of *H. influenzae* strains (n = 50).

AMR profile *	(*n*) (%)
AMP, AMC, CAZ	2 (4)
AMP, CTX, CAZ	1 (2)
AMP, CAZ, IPM	1 (2)
AMP, AMC	6 (12)
AMP, CAZ	2 (4)
AMP, CTX	1 (2)
AMP, IPM	2 (4)
CTX, CAZ	1 (2)
AMC	1 (2)
AMP	21 (42)
CAZ	3 (6)
$	9 (18)

* AMP: ampicillin; AMC: amoxicillin-clavulanate; CTX: cefotaxime; CAZ: ceftazidime; IPM: imipenem; $: susceptible to ampicillin, amoxicillin-clavulanate, piperacillin-tazobactam, cefotaxime, ceftazidime, ceftriaxone, cefepime, imipenem and meropenem.
